# Complete Genome Sequence of the Endophytic Bacterium Chryseobacterium indologenes PgBE177, Isolated from Panax quinquefolius

**DOI:** 10.1128/MRA.01234-18

**Published:** 2018-10-11

**Authors:** Chi Eun Hong, Jang Uk Kim, Jung Woo Lee, Kyong Hwan Bang, Ick-Hyun Jo

**Affiliations:** a Department of Herbal Crop Research, National Institute of Horticultural and Herbal Science, Rural Development Administration, Eumseong, Republic of Korea; Georgia Institute of Technology

## Abstract

Chryseobacterium indologenes PgBE177, isolated from the root tissue of a 4-year-old Panax quinquefolius plant, showed antagonistic activity against Pseudomonas syringae pv. tomato DC3000, a bacterial pathogen. Here, we report the whole-genome sequence of C. indologenes PgBE177. The bacterium contains bacteriocin gene clusters and has the potential to stimulate plant growth.

## Abstract (precis)

Chryseobacterium indologenes PgBE177, isolated from the root tissue of a 4-year-old Panax quinquefolius plant, showed antagonistic activity against Pseudomonas syringae pv. tomato DC3000, a bacterial pathogen.

## ANNOUNCEMENT

The Chryseobacterium genus includes important bacteria associated with plants (1, 2). Chryseobacterium species are yellow-pigmented Gram-negative bacteria and exist as endophytes in several plants (3) as follows: C. hispalense, with plant growth-promoting properties, was isolated from a rainwater pond in an olive plant nursery (1), and C. indologenes AM2 was isolated from root nodules of Vigna radiata (3). Besides its plant growth-promoting trait, C. indologenes AM2 exhibited nitrogen fixation activity, 1-amiocyclopropane-1-carboxylate (ACC) deaminase activity, and *nifH* gene expression (3). Recently, we isolated endophytic bacteria from various tissues of Panax quinquefolius. The 4-year-old plants were collected from the National Institute of Horticultural and Herbal Science of the Rural Development Administration in Chungbuk Province, Republic of Korea (127°45′13.14″E, 36°56′36.63″N). The harvested samples were separated into root, stem leaf, and flower stalk tissues and were homogenized in 1 ml sterilized water by using a TissueLyzer after disinfection with 70% ethanol, 12% NaOCl, and sterilized water. We obtained single colonies from the LB plate at room temperature after spreading bacterial suspensions. Among these bacteria, a root-inhabiting strain, PgBE177, was identified as C. indologenes via 16S rRNA gene sequencing analysis (GenBank accession number MH211268).

To elucidate the functional significance of C. indologenes PgBE177, we determined its genome sequence using the PacBio RS II system (Chunlab, Inc.). For bacterial genomic DNA extraction, bacterial cells were incubated with Chelex 100 resin (catalog number 143-2832; Bio-Rad, CA, USA) and proteinase K at 65°C for 30 min and then boiled at 100°C for 10 min. After centrifugation at 10,000 × *g* for 10 min, the supernatants containing bacterial genomic DNA were obtained. Sample preparation was based on a SMRTbell template preparation kit (Pacific Biosciences, Menlo Park, CA, USA), following the manufacturer’s protocol. A BluePippin size selection system was used to size select the 20-kb DNA template. Following DNA size selection, the libraries were sequenced using the PacBio RS II instrument with P6-C4 chemistry (Pacific Biosciences) in an 8-well single-molecule real-time (SMRT) cell (version 3). The assembly process was performed with PacBio SMRT Analysis 2.3.0 using the Hierarchical Genome Assembly Process 2 (HGAP2) pipeline. The genome size was 5,008,467 bp, with a GC content of 36.4% and *N*_50_ value of 5,008,467 bp. The genome of C. indologenes was completely assembled, and the depth of coverage was 211.23-fold.

The protein-coding sequences (CDS) were predicted using Prodigal 2.6.2 (4). Genes coding for tRNAs were searched using tRNAscan-SE 1.3.1 (5). Using a covariance model, the Rfam 12.0 database was searched for rRNAs and other noncoding RNAs (6). The CDS were classified into groups based on their roles, with reference to orthologous groups (EggNOG 4.5; http:/eggnogdb.embl.de) (7). For further functional annotation, the genome sequence was analyzed using the RAST and antiSMASH 4.2.0 annotation engines (8, 9). A total of 4,511 CDS were identified, along with 86 tRNA genes and 18 rRNA genes. The 9 gene clusters, including 1 bacteriocin, 3 terpene gene clusters, and 1 siderophore type, were predicted by antiSMASH in the C. indologenes PgBE177 genome sequence. Bacteriocins are antimicrobial peptides produced by bacteria, which can inhibit the growth of other bacteria (10). These antimicrobial compounds might be useful for inhibiting bacterial phytopathogen growth. Although a previous study reported that C. indologenes AM2 cannot produce siderophores, our analysis revealed a gene cluster for siderophore biosynthesis in the C. indologenes PgBE177 genome sequence (3). Furthermore, the auxin biosynthesis subsystem associated with plant growth was detected in the C. indologenes PgBE177 genome. Based on our results, C. indologenes PgBE177 can potentially stimulate plant growth-promoting activity. This genome information will provide an overview of the genetic and functional characteristics of C. indologenes PgBE177.

### Data availability.

The C. indologenes PgBE177 genome sequence has been deposited in GenBank with the accession number SRR7691104 under BioProject number PRJNA485193.
